# Series 1: Behind the Spread: A Scoping Review of Risk Factors for Exposure to *Mycobacterium tuberculosis*

**DOI:** 10.3390/tropicalmed11020058

**Published:** 2026-02-19

**Authors:** Sonia Menon, Anthony D. Harries, Riitta A. Dlodlo, Gisèle Badoum, Mohammed F. Dogo, Olivia B. Mbitikon, Pranay Sinha, Yan Lin, Jyoti Jaju, Aung Naing Soe, Anisha Singh, Bharati Kalottee, Kobto G. Koura

**Affiliations:** 1 International Union Against Tuberculosis and Lung Disease, 75001 Paris, France; sonia.menon.consultant@theunion.org (S.M.); adharries@theunion.org (A.D.H.); rdlodlo@theunion.org (R.A.D.); gisele.badoum.consultant@theunion.org (G.B.); fall.dogo.consultant@theunion.org (M.F.D.); olivia.mbitikon.consultant@theunion.org (O.B.M.); psinha@bu.edu (P.S.); ylin.consultant@theunion.org (Y.L.); jyoti.jaju@theunion.org (J.J.); anisha.singh@theunion.org (A.S.); bharati.kalottee@theunion.org (B.K.); 2 Epitech Research, 1160 Auderghem, Belgium; 3 Department of Clinical Research, Faculty of Infectious and Tropical Diseases, London School of Hygiene and Tropical Medicine, London WC1E 7HT, UK; 4 Unité de Formation et de Recherche en Sciences de la Santé (UFR/SDS), Université Joseph KI-ZERBO, Ouagadougou P.O. Box 7021, Burkina Faso; 5 Service de Pneumologie, Centre Hospitalier et Universitaire de Yalgado Ouédraogo (CNHU-YO), Ouagadougou P.O. Box 7022, Burkina Faso; 6 Section of Infectious Diseases, Boston University Chobanian and Avedisian School of Medicine, Boston, MA 02118, USA; 7 Boston Medical Center, Boston, MA 02118, USA; 8 Unité Mixte de Recherche MERIT (UMR261), Université Paris Cité, Institut de Recherche pour le Développement (IRD), 75006 Paris, France

**Keywords:** *Mycobacterium tuberculosis* exposure, climate change, air pollution, occupational health, homelessness, migration, multi-sectoral approach

## Abstract

Background: Tuberculosis (TB) remains a major global health challenge, with transmission influenced by the incidence of contagious people with TB, the duration of infectivity, and the probability of contact with susceptible individuals. This review synthesizes recent evidence on established and emerging risk factors influencing TB transmission, particularly in light of global trends such as migration, urbanization, and demographic shifts, to guide future prevention and control strategies. This scoping review maps and synthesizes evidence from systematic reviews on risk factors for *Mycobacterium tuberculosis* exposure. Methods: A preliminary general literature search was conducted in PubMed on 25 August 2024, using the keywords “tuberculosis,” “risk factors,” and “systematic review.” A subsequent targeted search focused on systematic reviews published since 2000 that examined social and environmental determinants of exposure to *M. tuberculosis* identified in the general search. Original research and reviews spanning pre-2000 were excluded. Data extraction and synthesis followed PRISMA-ScR guidelines. Results: Of the 344 systematic reviews identified, 14 met the eligibility criteria, reporting on key risk factors contributing to the incidence of contagious people with TB, the duration of infectivity, and the probability of contact. These risk factors included homelessness, migration, occupational exposure, urbanization, climate change, and air pollution. The findings emphasize the complex interrelated role of social and environmental determinants in driving TB transmission. Conclusion: This review highlights the need for a multi-sectoral approach to TB, as climate change, air pollution, overcrowding, stigma, and limited healthcare access exacerbate established risks related to poverty. Effective prevention and control require targeted interventions that address these interconnected factors.

## 1. Introduction

Tuberculosis (TB) continues to be a critical global health issue, with exposure to *Mycobacterium tuberculosis* (*M. tuberculosis*) driven by three core dynamics: the incidence of contagious people with TB, the duration of infectivity, and the probability of contact between contagious individuals and susceptible populations [1]. In classical TB epidemiology, this transmission-focused paradigm conceptualizes exposure as a function of these three components, even though empirical studies and surveillance systems most often operationalize transmission using TB disease incidence as a proxy.

Historically recognized as a “social disease,” [2] TB’s epidemiology is profoundly influenced by socioeconomic factors. Notably, inadequate housing, characterized by overcrowding and poor ventilation, elevates the incidence of TB [3]. Global trends reveal stark disparities, with lower-middle and upper-middle income countries (LMICs) consistently exhibiting higher rates of TB compared to high-income countries [4]. Regional variations further emphasize the influence of economic and geographic factors on *M. tuberculosis* transmission [4].

Since 2000, global trends such as migration and rapid urbanization have continued to reshape the TB transmission landscape. According to the United Nations, the urban population is set to increase by almost 600 million by the year 2030, reaching a total of 5.2 billion. By mid-2023, approximately 4.6 of the more than 8 billion people worldwide lived in towns or cities, representing 57% of the global population [5]. Urban growth, particularly in Asia and Africa, will intensify TB risks due to higher contact rates, with Africa’s urban population projected to surge from 414 million to over 1.2 billion by 2050, while that of Asia will soar from 1.9 billion to 3.3 billion [6]. Migration and homelessness further increase susceptibility as these groups often face overcrowded and unstable conditions with limited healthcare access. As a corollary, adverse conditions may hamper healthcare delivery, further exacerbating the risk of TB infection among marginalized populations [7].

Furthermore, environmental factors such as air pollution and climate change are also emerging influences on TB spread. Climate change may increase TB incidence by promoting conditions that favor *M. tuberculosis* survival, potentially extending the duration of infectivity [8]. To inform future prevention and control strategies, this scoping review synthesizes evidence from systematic reviews to update established social, occupational, and environmental risk factors for exposure to *M. tuberculosis* and identify emerging factors impacting TB incidence, duration of infectivity, and probability of contact since 2000. This review constitutes the first paper in a structured series examining the natural history of tuberculosis. The present manuscript focuses specifically on social, occupational, and environmental risk factors for exposure to *M. tuberculosis*.

## 2. Materials and Methods

### 2.1. Search Strategy and PEOS Questions

On 25 August 2024, we conducted an initial broad literature search in PUBMED using the keywords “tuberculosis” AND “exposure” to identify potential indirect social and environmental risk factors associated with TB transmission and infection. Titles and abstracts were screened in accordance with the research framework, which subsequently informed the targeted follow-up searches. This search included all study types published from January 2000 onward and applied no language restrictions to ensure comprehensive coverage. We registered our protocol with OSF https://osf.io/gn74s/overview (accessed on 22 December 2024).

Based on the risk factors identified in this initial search, we conducted a targeted follow-up search focused on each specific factor in relation to exposure to *M. tuberculosis*. This first phase was restricted to systematic reviews published between January 2000 and 24 August 2024, in order to synthesize high-quality and up-to-date evidence. Additionally, reference lists of pertinent systematic reviews were examined to identify any additional relevant reviews/studies.

The primary research question guiding this scoping review was: What are the current social and environmental risk factors for exposure to *M. tuberculosis*? To structure the review, the following PEOS question was formulated to systematically scope relevant literature.

*Population:* Populations at risk for TB.
*Exposure:* Social, occupational, and environmental risk factors influencing TB incidence and exposure.
*Outcome:* TB incidence, duration of infectivity, and probability of contact.
*Study design:* Systematic reviews published within the specified timeframe (2000–2024).

### 2.2. Inclusion and Exclusion Criteria

Only systematic reviews, irrespective of the language, focusing on social, occupational, and environmental determinants of TB from 2000 were included in this review. Original research, commentaries, editorials, and other types of literature, and systematic reviews that only included studies before 2000 were excluded.

### 2.3. Data Extraction, Synthesis, and Reporting

Data regarding risk factors influencing *M. tuberculosis* exposure from the included reviews were independently extracted by two reviewers. Key findings were synthesized into categories which emerged organically from the data based on the reviewed literature, exploring their interrelatedness with TB incidence, duration of infectivity and probability of exposure. The scoping review adhered to PRISMA-ScR (Preferred Reporting Items for Systematic Reviews and Meta-Analyses, Extension for Scoping Reviews) guidelines [9]. The PRISMA-ScR checklist is available in the Supplementary Materials Table S1.

### 2.4. Use of Non-Stigmatizing Language

We have adopted non-stigmatizing and person-centered terminology throughout this manuscript when referring to TB and individuals affected by it. Terms such as “people with TB,” have been used instead of “TB patients” or “TB cases”. We also use “TB infection” instead of “latent TB infection” and refer to “TB disease” rather than “active TB,” except where legacy terms are needed for clarity in cited literature. This linguistic approach aims to reduce stigma, promote respect and dignity, and reflect the evolving norms in global TB research and practice [10].

## 3. Results

### 3.1. PRISMA Flow Diagram and Systematic Review Characteristics

**Figure 1:**
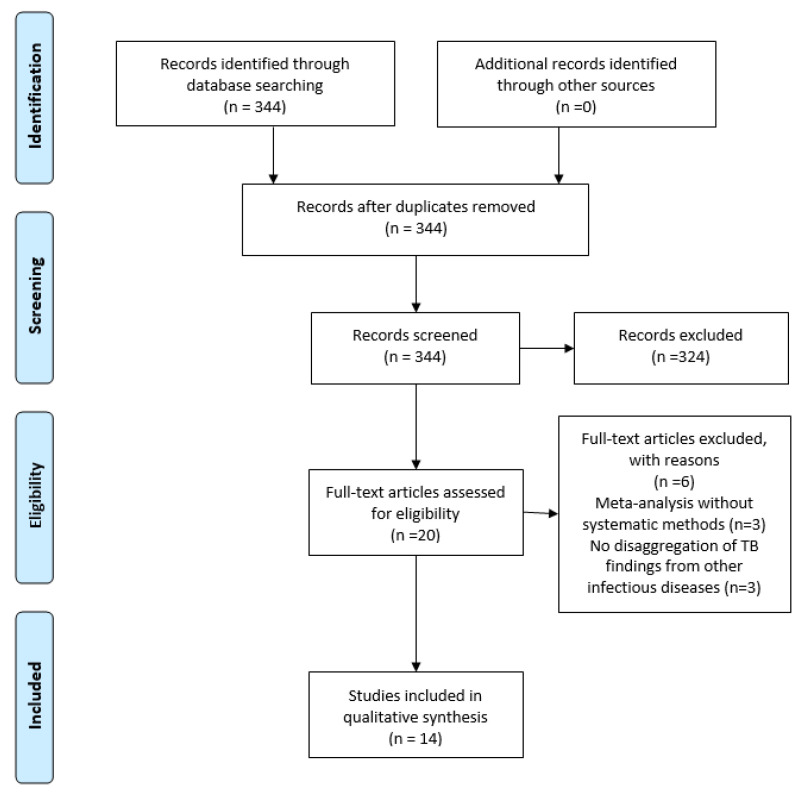
PRISMA Flow Diagram.

We conducted a systematic search to identify reviews addressing TB in relation to key social and environmental determinants. The search strategy included terms for each domain (homelessness, migration, occupational health, urbanization, climate change, and air pollution) combined with terms for TB and for systematic reviews. This yielded a total of 344 systematic reviews, categorized as follows: 27 on homelessness and TB, 21 on migration and TB, 111 on occupational health and TB, 33 on urbanization and TB, 41 on climate change and TB, and 23 on air pollution and TB. After screening, 14 reviews met the eligibility criteria and were included in this scoping review (see Figure 1 for the PRISMA flow diagram and Table 1 for the Systematic Review Characteristics).

### 3.2. Social Risk Factors Increasing TB Incidence in the Community

#### 3.2.1. Homelessness and Housing

Homelessness and inadequate housing increase the likelihood of exposure of susceptible populations to contagious people with TB in crowded settings. Three reviews highlight the complex, intersecting challenges faced by individuals in unstable living conditions.

A systematic review by Gioseffi et al. (2022) [11] analyzed 16 studies published between 2014 and 2020. This review explored the intersection of TB and HIV among homeless individuals, emphasizing that the duration of homelessness is associated with a higher incidence of significant vulnerability factors for TB, including substance use. Homeless individuals also frequently face stigma and dehumanization, which act as barriers to accessing healthcare services. These barriers contribute to prolonged periods of infectivity, which, in turn, increase the likelihood of TB transmission within homeless settings. Furthermore, Hino et al. (2021) [12] systematic review, which included seven studies, highlighted the poorer treatment outcomes in homeless individuals with TB compared to those in stable housing. This disparity in outcomes was linked to factors such as substance use, especially alcohol and drug use, as well as comorbid conditions such as HIV, which exacerbate the probability of being exposed to contagious TB.

Similarly, Lee et al. (2022) [3] examined the relationship between inadequate housing and TB across 26 studies published between 2011 and 2020. Multiple outcomes were explored, including exposure to *M. tuberculosis*. The review found that exposure risk was more closely associated with housing affordability than with housing quality, with patterns varying by countries’ economic status.

#### 3.2.2. Migration

Migration driven by conflict, economic instability, and climate-related disasters has led to overcrowded living conditions, such as refugee camps and urban slums, where the likelihood of contact with people with contagious TB and the duration of infectivity are increased. Two systematic reviews explored the relationship between migration and risk of exposure.

In their 2021 systematic review, using a cut-off of 2010, Jackson et al. (2021) [13] investigated the epidemiological profile of TB among migrants from high-incidence countries compared to non-migrants in low- to medium-incidence areas based on 93,235 people with TB. Their findings revealed that migrants exhibit markedly higher rates than non-migrants of multidrug-resistant TB (MDR-TB), clustered cases, and HIV co-infections, along with lower treatment success rates. Specifically, the pooled odds ratio (OR) for MDR-TB among migrants was 3.91 (95% CI: 2.98–5.14). Additionally, a 2020 systematic review by Proença et al. (2020) [14], which examined studies published between 2000 and 2017, and included data on 537,218 individuals with TB, found that refugees and asylum seekers face a substantially elevated TB risk. The review reported an average TB prevalence of 1331 per 100,000 inhabitants and a 37% prevalence rate for TB infection.

#### 3.2.3. Urbanization

Rapid urbanization has led to the proliferation of slum settlements with living conditions that facilitate the probability of contact with individuals with contagious TB, and a long duration of infectivity. A systematic review and meta-analysis conducted by Noykhovich et al. (2019) [15] revealed that the odds of sputum smear-positive TB among slum residents were nearly three times higher than national averages, with an OR of 2.96 (95% CI: 2.84–3.09). This elevated risk was exacerbated by factors such as overcrowding, inadequate sanitation, and limited access to healthcare. Among the 11 studies reporting the incidence of smear-positive TB with prevalent TB-HIV coinfection in the community, the pooled OR for slum residents was 2.48 (95% CI: 2.34–2.63; *p* < 0.01).

### 3.3. Occupational Exposure

#### 3.3.1. Healthcare Workers and Correctional Facility Workers

Workers in congregate settings, including healthcare workers (HCWs) and correctional facility workers, are at an elevated risk of exposure to contagious people with TB within a confined setting, compared to the general population, despite ongoing efforts to implement infection control and prevention measures to reduce nosocomial TB transmission. Three systematic reviews reported on this association.

Uden et al. 2017 [16] reviewed 21 studies (5 from high-income countries) which included a total of 30,961 HCWs. Their findings indicated that HCWs are more susceptible to contagious TB, with an estimated TB incidence of 97 cases per 100,000 HCWs per year, ranging from 42 to 4393 cases per 100,000 HCWs per year. Notably, HCWs were found to be 2.94 (CI: 95% 1.67–5.19) times more likely to develop TB disease than individuals in the general population, thus increasing exposure of other susceptible HCWs to contagious cases of TB by both patients and other HCWs. In a subsequent systematic review carried out by Silva et al. 2022 [17], which analyzed the incidence of TB among HCWs across 24 studies, annual incidence rates spanned from 1.4% to 11.4%., reinforcing the notion that occupational risk factors, such as direct patient contact and prolonged exposure to infectious individuals, play a critical role in the increased incidence of TB among HCWs. The review underscored that gaps persist in the understanding of TB epidemiology among HCWs, particularly regarding specific contributing factors. Grenzel et al. (2018) [18] conducted a systematic review of studies examining TB infection and TB disease among correctional facility workers. The pooled prevalence of TB infection in this population was 26% (95% CI: 12–42%) with an incidence of 2% (95% CI: 1–3%). In high-burden countries, the prevalence of TB infection increased to 44% (95% CI: 12–79%). Notably, all reported episodes of TB disease occurred in low-burden countries, with incidence rates ranging from 0.61 to 450 people diagnosed per 10,000 workers per year. Identified risk factors for TB infection included longer duration of employment, older age, being born in a high-incidence country, current tobacco use, contact with incarcerated individuals, and prior BCG vaccination.

#### 3.3.2. Silicosis Exposure

Occupational hazards in various industries, including mining, construction, pottery, marble stone production, and sand extraction, further compound the risk of exposure to *M. tuberculosis* [24]. Workers in these sectors are often subjected to poorly ventilated environments and prolonged contact with respiratory irritants, notably silica dust [24]. Jamshidi et al. (2023) [19] conducted a systematic review and meta-analysis examining the association between silica dust exposure and TB risk. Based on four cohort studies and a predominantly male population (90.63%), the pooled risk ratio for TB among individuals with silicosis or silica exposure was 1.35 (95% CI: 1.18–1.53), indicating a significantly increased risk.

### 3.4. Environmental Risk Factors

#### 3.4.1. Climate Change

Anthropogenic greenhouse gas emissions pose significant respiratory health risks, rendering climate change a critical global public health challenge that impacts TB burden in multifaceted ways [8]. Recent decades have introduced new risks for TB as climate change alters environmental conditions affecting *M. tuberculosis* viability. Rising temperatures, humidity, and shifting precipitation patterns may enhance the survival of the bacteria in the environment, increasing exposure potential [25]. Two systematic reviews report on TB incidence related to seasonal variations and climate change.

In a systematic review by Tedijanto et al. (2018) [20], 57 studies were analyzed to investigate seasonal variations in TB incidence. Of these, 49 studies reported that TB incidence peaked in the spring or summer and reached its lowest levels in late fall or winter. Several hypotheses were proposed to explain this variation, including host immune responses influenced by changes in vitamin D or melatonin, co-infections with seasonal respiratory viruses, increased seasonal indoor crowding, and environmental factors such as humidity and temperature affecting *M. tuberculosis* survival. Kharwadkar et al. (2022) [21] linked climate change to TB risk factors outlined in the Global Tuberculosis Report 2021, such as poverty, undernutrition, and overcrowding. Of 53 studies, the majority showed positive associations between climate change and TB risk factors, especially in low-resource settings where poverty and undernutrition are exacerbated by climate-related events.

#### 3.4.2. Air Pollution

Ambient fine Particulate Matter (PM) has become a critical environmental health risk, aggravated by urbanization, transportation emissions, and energy demands. Due to their small size, PM particles are especially toxic, promoting inflammatory responses and impairing immune defenses [26] which may increase susceptibility to respiratory infections, including TB. Two systematic reviews emphasize the strong link between air pollution and TB.

Dimala et al. (2022) [22] reported that for every 10 μg/m^3^ increase in PM_2.5_, there was a significant 12% rise in pulmonary TB (PTB) incidence. Similarly, Xiang et al. (2021) [23] in a systematic review of 17 studies found that long-term exposure to PM_10_ (RR = 1.058, 95% CI: 1.021–1.095), SO_2_ (RR = 1.016, 95% CI: 1.001–1.031), and NO_2_ (RR: 1.010, 95% CI: 1.002–1.017) was consistently associated with higher TB incidence, reinforcing the role of air pollutants in TB transmission.

## 4. Discussion

### 4.1. Summary of Results

This review synthesized evidence on selected social, environmental, and occupational risk factors associated with TB incidence generated by 14 systematic reviews. Evidence from systematic reviews suggests that homelessness increases the risk of TB due to drug use, HIV co-infection, and treatment failure in shelters, resulting in heightened exposure of a susceptible homeless population to TB disease in congregate settings. Also, exposure to *M. tuberculosis* appears driven more by housing affordability than housing quality, with economic disparities limiting access to stable, affordable housing. As a corollary, this often forces individuals into overcrowded or transient settings, creating conditions that facilitate TB transmission. Additionally, homeless individuals with TB face poorer treatment outcomes, further worsened by substance use and comorbidities such as HIV infection. As a result, poorer treatment outcomes that include treatment failure and the need for further treatment regimens further prolong TB infectivity in congregate homeless settings, increasing exposure risk for this already vulnerable population. In high-income countries, migrant populations, particularly foreign-born individuals, face elevated TB risks due to latent infections acquired in high-burden areas, compounded by barriers to treatment. As a result, susceptible migrants may be further exposed to newly contagious people with TB within their respective communities. Certain occupations are associated with heightened vulnerability to exposure to *M. tuberculosis* due to a higher incidence of persons with contagious TB in health facilities and other congregate settings and high-risk environments, such as correctional facilities and industries involving silica exposure. Finally, air pollution, particularly increased PM_2.5_ levels, correlates with higher TB incidence, highlighting the need for multisectoral strategies targeting both air quality and TB transmission.

### 4.2. Public Health Impact

Over the past two decades, the complex interplay of social and economic factors has created the stage to substantially elevate TB incidence by increasing exposure to *M. tuberculosis*. Overcrowded slums, inadequate housing, and limited access to healthcare services increase contact with individuals with unresolved TB, while migration-driven economic instability exacerbates overcrowding and homelessness, further fueling TB transmission. Research suggests that migrants from high-prevalence regions, that include Syria, Iraq, and Afghanistan, exhibit higher rates of MDR-TB and clustered cases due to reactivated latent infections acquired in their regions of origin, along with lower treatment success rates [27]. Migration often funnels individuals into urban slums with inadequate healthcare infrastructure, particularly unregulated clinics, where diagnostic delays and inconsistent treatment prolong infectiousness. Additionally, stigmatization surrounding TB further discourages positive healthcare-seeking behavior and thus renders TB control efforts in vulnerable communities challenging. The unprecedented influx of refugees and asylum seekers results in an increased exposure to people with TB within their respective migrant communities. Further complicating these challenges are population growth and rapid urbanization in LMICs, which are likely to increase TB recurrence rates, intensifying exposure and posing significant challenges to TB control in vulnerable communities. These factors strain already limited resources and contribute to the expansion of urban slum populations, who remain underserved by formal healthcare systems. Together, these interconnected factors amplify TB transmission risks, prolong infectivity, and increase exposure, underscoring the urgent need for public health interventions that address the social determinants of TB in these high-risk populations. Special attention is needed for asylum seekers, who face elevated TB rates due to the frequent collapse of essential infrastructure in their countries of origin, including healthcare and education, in the wake of civil unrest. Tailored interventions and systematic surveillance are essential to control TB within this population, ultimately improving long-term treatment outcomes.

Environmental factors, including air pollution and climate change, amplify the risk of TB by interacting with social determinants of health. Consequently, these factors are likely to contribute to an increased incidence of contagious people with TB, thereby elevating the risk of exposure among vulnerable populations. Chronic exposure to fine particulate matter (PM_2.5_) diminishes respiratory health, especially among populations living in overcrowded urban slums or experiencing homelessness. These conditions increase susceptibility to exposure to *M. tuberculosis*, facilitating its transmission within conglomerate settings. Whilst air quality improvements may be observed, increasing population growth may still increase exposure to pollutants, thereby elevating TB incidence rates. In addition, climate change may further compound these risks by driving migration, often from high-prevalence regions, leading to urban overcrowding. Adding to this complexity, risk factors for TB, including smoking, further impair respiratory health and are expected to play a salient role. In sub-Saharan Africa, where over 77 million adults currently smoke, this number is projected to rise significantly to 413 million by 2100 [28]. Considering the high population density in many areas of the region, the anticipated increase in smoking prevalence is likely to exacerbate the incidence of contagious TB and amplify the probability of contact with susceptible populations in densely populated settings.

Thus, the interplay of these environmental and social determinants underscores the necessity for the interconnected risk factors of TB to be addressed using a multi-sectoral public health approach. This approach should include expanding access to affordable housing to reduce overcrowding, a key driver of TB transmission risk; enhancing healthcare access; and redesigning urban environments to alleviate congestion, all of which are vital components of TB control strategies. Enforcing rigorous pollution control policies and climate adaptation strategies is also essential to mitigate TB risks.

Additionally, to reduce exposure to *M. tuberculosis*, regular screening for early detection of TB infection should be enforced in high-risk occupational settings, such as mining, healthcare facilities, and correctional institutions, where staff face prolonged exposure to silica dust or infectious aerosols.

Importantly, these risk factors rarely operate in isolation. Evidence synthesized in this review suggests that exposure to *M. tuberculosis* is often driven by the co-occurrence and interaction of social, occupational, and environmental determinants. Rapid urbanization frequently concentrates populations in informal settlements where overcrowding, poor ventilation, and elevated levels of ambient air pollution coexist, creating conditions that increase both the probability of contact with contagious individuals and the duration of infectivity. Similarly, migration and homelessness often intersect with stigma, precarious living conditions, and limited access to healthcare services, contributing to diagnostic delays and prolonged exposure within vulnerable communities. In occupational settings, long-term exposure to silica dust may interact with climate-related stressors and underlying socioeconomic vulnerabilities, potentially increasing susceptibility to TB and amplifying transmission risks. Together, these intersecting pathways highlight that TB exposure reflects a systemic accumulation of risks, rather than the effect of single determinants acting independently.

While this scoping review does not formally model mediating or moderating effects, it provides a structured overview of how multiple determinants converge across settings, thereby laying the groundwork for future analytical and modelling studies focusing on interaction mechanisms.

### 4.3. Research Gaps

Beyond the risk factors identified in the included systematic reviews, emerging evidence from recent primary studies suggests additional interactions that may further shape tuberculosis exposure and transmission dynamics. Emerging multicentric observational evidence from recent primary studies, not yet incorporated into existing systematic reviews, suggests that long-term exposure to PM_2.5_, combined with aging, may lead to an increased incidence of TB disease through reactivation [29]. However, the extent of this intersection remains poorly characterized. Aging weakens immune resilience, particularly within the respiratory system, potentially heightening susceptibility to PM_2.5_-related impacts on TB. In areas with poor air quality and large aging populations, this combination may accelerate the progression of TB infection to active disease, thereby increasing the incidence of contagious TB. More research on this interaction is crucial for designing TB prevention strategies targeted at older, vulnerable populations in polluted areas.

A key research gap lies in understanding the causal mechanisms behind the seasonal variation in TB incidence [20]. While several hypotheses suggest potential explanations, such as host immune responses influenced by changes in vitamin D or melatonin, co-infections with seasonal viruses, indoor crowding, and environmental factors such as humidity and temperature, further studies are needed to clarify the specific pathways through which these factors affect *M. tuberculosis* survival and transmission.

More broadly, TB transmission dynamics are shaped by a complex interplay of socioeconomic, environmental, demographic, and climate factors, such as temperature fluctuations, wind patterns [30] and exposure to PM_2.5_. These interactions result in varying TB risks across populations, with urban areas facing heightened transmission due to overcrowding, poor ventilation, and pollution. As urbanization and demographic shifts alter exposure patterns, elucidating these interdependencies is crucial for TB control. Developing mathematical models to simulate these effects across different demographics and population densities could help inform targeted prevention strategies. Furthermore, AI-driven techniques may improve these models by capturing non-linear relationships and adapting to real-time data.

Another key research gap emerging from this synthesis concerns the limited empirical evidence quantifying how interacting social, environmental, and occupational risk factors jointly shape TB exposure and transmission dynamics across different settings and populations.

Finally, further studies are needed to explore how stigma varies across different cultural contexts and to identify effective tailored interventions to reduce its impact, particularly in high-burden settings and among vulnerable populations.

### 4.4. Strengths and Limitations

Our scoping review has several strengths. The search strategy ensured an exhaustive literature review by incorporating a wide range of relevant keywords, allowing for the exploration of multiple dimensions of TB risk. Additionally, the absence of language restrictions in the search strategy minimized potential biases, ensuring that relevant evidence from non-English sources was included.

However, some individual-level risk factors, such as smoking, alcohol consumption, and malnutrition, influence TB risk and treatment outcomes, and consequently affect the incidence rate of TB across multiple stages of its natural history. These factors are only briefly addressed in this review, as their detailed analysis is reserved for subsequent reviews focusing on risk factors for progression from infection to TB disease and TB treatment outcomes.

A key methodological challenge is that the incidence of contagious individuals cannot be directly measured in most studies, as routine surveillance and published systematic reviews predominantly report TB disease incidence without consistently distinguishing contagious from non-contagious forms. As a result, TB disease incidence is commonly used as an operational proxy for exposure and transmission risk.

This scoping review relied on PubMed as the primary database, which may have resulted in the omission of relevant systematic reviews indexed exclusively in other databases, particularly those covering environmental or social science literature.

Whilst the scoping review’s focus on systematic reviews provides a high-level synthesis of evidence enabling a more robust and contemporary assessment of established and emerging social and environmental risk factors for exposure to *M. tuberculosis*, the reliance on systematic reviews may not capture data from primary studies and emerging evidence that may not yet be encapsulated in the existing reviews. Additionally, the variability in the quality of the studies included in the systematic reviews may affect the reliability of the synthesized findings. Importantly, our review did not examine the intensity of TB infectivity, such as the differential transmission risk posed by smear-positive versus smear-negative index cases, as this key driver of TB transmission falls outside the scope of our review, which was focused on socio-economic risk factors. Finally, the exclusion of reviews spanning pre-2000 studies may have resulted in an incomplete assessment of risk factors.

## 5. Conclusions

TB remains a critical global health challenge, driven by a complex interplay of social, environmental, and occupational factors that increase the incidence of contagious TB, prolong infectivity, and elevate exposure risk. Emerging threats, such as climate change and air pollution, may exacerbate established risks such as homelessness, inadequate housing, smoking, and exposure among workers in congregate settings, as well as migrants and refugees. Additionally, stigma and barriers to care further fuel transmission.

As highlighted by the interactions observed between social, environmental, and occupational risk factors in this review, reducing exposure to *M. tuberculosis* requires coordinated action beyond the health sector alone. These findings are consistent with the WHO multisectoral frameworks, including the Multisectoral Accountability Framework for TB [31]. A multi-sectoral poverty reduction-focused integrated approach, incorporating targeted interventions and addressing TB as a climate-sensitive disease, is crucial to reduce exposure to *M. tuberculosis* and to inform effective prevention strategies.

## Figures and Tables

**Table 1:** Systematic review characteristics included in the current scoping review.

Author of Systematic Review	Date Range of Eligible Studies	Number of Studies	Objective	Methodological Assessment	Conclusion
Lee et al. (2022) [3]	2011–2020	26 studies (Asia, Americas, Africa, and Europe)	Identify the impact of inadequate housing on TB and categorize exposure based on affordability and quality.	Joanna Briggs Institute checklists: (21 had low risk and 2 had intermediate risk).	The steps in TB development and their consequences were more strongly associated with housing affordability than quality. Public health interventions should prioritize both affordable housing and quality improvements for residents of inadequate housing and the homeless.
Gioseffi et al. (2022) [11]	2014–2020	16 studies (Asia, Americas, Europe)	Analyze and compile social, individual, and programmatic vulnerability factors for TB and HIV in homeless individuals.	Most studies had biases related to group recruitment, confounding factors, mitigation strategies, and incomplete follow-ups. Few acknowledged limitations, and those that did had methodological and reporting flaws.	All studies highlighted stigma and dehumanization as key barriers to health service access for homeless individuals. Homelessness increases the risk of chronic and infectious diseases, with survival priorities like safety and food taking precedence over health. These findings can inform future research and strengthen public health and social policies addressing TB and HIV in the homeless.
Hino P et al. (2021) [12]	2005–2016	7 studies (number of participants unknown), North America, Asia, Europe, Latin America	Analyze evidence of the occurrence of TB in people living on the streets provided by the literature.	Not available.	Homeless individuals with TB experience worse treatment outcomes than the general population, often due to substance abuse and comorbidities like HIV. This highlights the need for further research not only on the disease’s prevalence in this vulnerable group but also on effective strategies for combating it.
Jackson et al. (2021) [13]	2010–2020	32 studies (25 in low TB incidence countries, while 7 in medium TB incidence countries (93,235 TB cases))	Synthesize evidence on key epidemiological differences in TB between migrants from high-incidence countries and non-migrants in low- to medium-incidence settings.	NIH risk of bias. No study achieved a low risk of bias score in all domains, with a median of 9 low risk domains out of a total of 13 domains assessed.	The epidemiology of TB differs between high-incidence migrants and non-migrants, with migrants showing higher odds for MDR-TB (OR:3.91; 95% CI: 2.98–5.14). A significant gap in the literature exists regarding specific data on migrant TB origins.
Proença et al. (2020) [14]	2000–2017	67 studies (537,218 active TB, with a single study evaluating 232,738 individuals)	Pool the prevalence of active and latent TB in refugees and asylum seekers through a systematic review and meta-analysis by country of origin and host continent.	STROBE checklist: Among the 33 cross-sectional and 32 cohort studies, only 13 and 11 respectively were considered high quality; 14 and 18 studies were of medium quality; and 6 cohort and 3 cross-sectional studies were considered low quality.	Active TB prevalence averaged 1331 per 100,000 (95% CI: 542–2384) and LTBI 37% (95% CI: 23–52%), both showing high heterogeneity (I^2^ > 98%). Prevalence varied more by country of origin than by host continent. Most studies reported routine screening, but many lacked critical data.
Noykhovich et al. (2019) [15]	1993–2017	22 studies (All of countries, excluding Iran, are or have been high-burden countries in the 21st century)	Assess the odds of the burden of TB in urban slums through a systematic review and meta-analysis.	Most studies reported no conflicts of interest, while 5 had undeclared conflicts. Nearly all described methods and sampling procedures, and all defined TB cases. Most acknowledged limitations, with some noting confounding factors or underestimated TB incidence.	Slum residents face significantly higher odds of TB compared to national rates, with a combined OR of 2.96 (95% 2.84–3.09) for smear-positive TB and 2.48 for TB-HIV coinfection (*p* < 0.01). Active case finding also showed elevated odds (OR 2.85; 95% CI: 2.71–2.99). Addressing TB in urban slums is critical to achieving the SDGs and the End TB Strategy by 2035.
Uden et al. (2017) [16]	2006–2016	21 studies (30,961 HCW) (Asia, Africa, Europe, South Africa)	Provide a pooled estimate of the occupational risk of LTBI and active TB among HCWs compared with the general population.	Strobe checklist: 6 studies were of low quality.	HCWs had higher risks than the general population, with an LTBI OR of 2.27 (1.61–3.20) and active TB incidence RR of 2.94 (95% CI: 1.67–5.19). HCWs remain at higher risk for LTBI and TB, emphasizing the need for improved infection control and screening programs.
Da Silva et al. (2022) [17]	2016–2019	24 studies (number of participants unknown)	Identify the prevalence and incidence of TB in HCWs.	Joanna Briggs Institute critical appraisal checklist (maximum 10 points) The mean quality score assessed was 8.1 points and 6 studies scored less than 6.	Annual TB incidence rates ranged between 1.4% to 11.4%. Risk factors included direct contact with TB patients and longer professional experience. TB remains a significant occupational hazard for HCWs, but gaps in understanding its epidemiology and risk factors persist.
Grenzel et al. (2018) [18]	1989–2017	15 studies (110,393 correctional facility workers; six countries; 82,668 active TB; 110,192 LTBI)	Investigated the magnitude of active and LTBI and associated risk factors among correctional facility workers.	Quality Assessment Tool for Observational Cohort and Cross-sectional Studies of the National Heart, Lung and Blood Institute (9 were assessed as good and 6 as fair quality).	Among correctional facility workers, pooled LTBI prevalence was 26% (95% CI: 12–42%) and incidence was 2% (95% CI: 1–3%), with prevalence reaching 44% (95% CI: 12–79%,) in high-burden countries. Active TB was reported only in low-burden countries (0.61–450 cases per 10,000 workers/year). Risk factors for LTBI included job duration, age, country of birth, smoking, prisoner contact, and BCG vaccination.
Jamshidi et al. (2023) [19]	1986–2023	7 studies (5884 participants); Europe, Sub-Saharan Africa, Asia	Evaluate the risk of TB in silicosis patients and individuals exposed to silica dust.	Based on the Newcastle-Ottawa Scale, the mean (standard deviation) score was 7.5 (1.29), suggestive of a high methodological quality and a low risk of bias.	Meta-analysis showed a pooled risk ratio of 1.35 (95% CI: 1.18–1.53), indicating an increased TB risk among silicosis patients and silica-exposed individuals. Silicosis and silica exposure significantly raise TB risk, highlighting the need for routine silicosis and TB screening in long-term silica-exposed populations, such as mine workers.
Tedijanto et al. (2018) [20]	Until 2017	57 studies (Only 11 out of 57 studies were from the Southern Hemisphere, with seven of these being from South Africa)	Identify demographic and ecological factors associated with the timing and magnitude of seasonal variation.	Not available.	TB incidence mostly peaked in spring/summer and was lowest in late fall/winter. The mean seasonal amplitude across 34 studies was 17.1% (range: 2.7–85.5%). Stronger seasonality was linked to younger patients, extrapulmonary disease, and higher latitudes. The model generally replicated observed seasonal variations but required significant risk variation for extreme values. TB showed spring peaks across countries, suggesting potential benefits from targeted mass screening interventions in spring.
Kharwadkar et al. (2022) [21]	2009–2021	53 studies (number of participants unknown)	To review epidemiological and prediction model studies that explore how climate change may affect the risk factors for TB.	NIH Quality Assessment Tool and PROBAST checklist: 15 studies were assessed to be of good quality and the remaining of fair quality.	Vote-counting revealed positive associations between climate change and risk factors for TB, including HIV (2/2 studies), diabetes (9/12), undernutrition (8/17), overcrowding (4/5), and poverty (12/15), but limited evidence for indoor air pollution (1/3). Climate change likely increases TB susceptibility by exacerbating underlying risk factors, especially in developing countries.
Dimala et al. (2022) [22]	2014–2020	24 studies (437,255 participants) mainly from Asian countries	Assess the extent to which selected air pollutants (PM_10_; SO_2_) are associated to TB incidence, hospital admissions and mortality.	National Health Institute/National Heart, Lung and Blood Institute: Twelve studies were of good quality, eleven of fair quality and one of poor quality.	A 10 μg/m^3^ increase in air pollutant concentration was significantly associated with higher PTB incidence for PM_2.5_ (pooled aRR = 1.12), PM_10_ (1.06), and SO_2_ (1.08), but not for CO, NO_2_, or O_3_. No associations were observed between air pollutants and PTB-related mortality or hospital admissions. Exposure to PM_2.5_, PM_10_, and SO_2_ increases PTB risk, though the evidence quality remains low, highlighting the need for further research.
Xiang et al. (2021) [23]	Until 2020	17 studies (number of participants unknown), China	Explore an association between air pollution exposure and TB incidence.	Newcastle-Ottawa scale. The methodological quality assessment results showed that the included articles were of reasonably good quality.	Long-term exposure to PM_10_, SO_2_, or NO_2_ is linked to increased TB risk, though the underlying biological mechanisms require further investigation.

## Data Availability

The data that support the findings of the study are available from one of the author (K.G.K.) upon reasonable request.

## Supplementary Materials

The following supporting information can be downloaded at: https://www.mdpi.com/article/10.3390/tropicalmed11020058/s1, Table S1: Preferred Reporting Items for Systematic reviews and Meta-Analyses extension for Scoping Reviews (PRISMA-ScR) Checklist—Risk factors for exposure to *Mycobacterium tuberculosis*.
